# A Preliminary Evaluation on Poly(3-Hydroxypropionic Acid) as a Novel Feed Additive on Growth Performance, Nutrient Digestibility, Selected Culturable Fecal Bacterial Counts, and In Vitro Fecal Fermentation in Growing Pigs

**DOI:** 10.3390/life16081326

**Published:** 2026-08-13

**Authors:** Vetriselvi Sampath, Kyejin Lee, Jeungyil Park, In Ho Kim

**Affiliations:** 1 Department of Animal Biotechnology, Dankook University, Cheonan 31116, Republic of Korea; vetri09@dankook.ac.kr (V.S.); jookyjin1234@dankook.ac.kr (K.L.); 2 Smart Animal Bio Institute, Dankook University, Cheonan 31116, Republic of Korea; 3 CJ CheilJedang, Yeongtong-Gu, Suwon-si 16495, Republic of Korea; jeungyil.park@cj.net

**Keywords:** body weight, dry matter digestibility, fecal N retention, in vitro fecal fermentation

## Abstract

Poly(3-hydroxypropionate) (P3HP) is a biodegradable member of the polyhydroxyalkanoate family that can be degraded into 3-hydroxypropionic acid, a three-carbon organic acid with potential biological activity. Although P3HP has attracted considerable interest for its microbial production and industrial applications, its potential as a feed ingredient in monogastric animals remains largely unexplored. Therefore, we aim to assess the effects of dietary P3HP supplementation on growth performance, nutrient digestibility, fecal N retention and apparent N absorption, selected culturable fecal bacterial counts, and in vitro fecal fermentation in growing pigs. A total of 100 [Duroc × (Landrace × Yorkshire) pigs; initial body weight 25.03 ± 1.70 kg] were assigned to four dietary treatments containing 0, 0.1, 0.2, and 0.3% P3HP for 6 weeks. At the end of the experimental period, dietary P3HP supplementation did not significantly (*p* > 0.05) affect final body weight, average daily gain, dry matter digestibility, nitrogen digestibility, apparent nitrogen absorption variables, or in vitro fecal fermentation in growing pigs but exhibited linear or quadratic trends (*p* < 0.1), with the most favorable numerical responses observed at the 0.2% inclusion level. In summary, although dietary P3HP supplementation did not significantly affect the principal measured outcomes, the favorable trends observed support further investigation of its potential as a novel feed additive for growing pigs.

## 1. Introduction

Growing concerns regarding the use of antibiotic growth promoters (AGPs) in livestock production have accelerated the search for alternative feed additives that can sustain animal performance while supporting intestinal health [1]. Consequently, considerable efforts have been directed toward identifying novel compounds that can deliver similar physiological benefits while contributing to sustainable animal production. Among the available alternatives, organic acids (OAs), particularly short-chain fatty acids (SCFAs) such as acetate, propionate, and butyrate, have attracted considerable interest because of their beneficial effects on intestinal epithelial integrity, nutrient utilization, gut microbial modulation, and growth performance [2]. Polyhydroxyalkanoates (PHAs) are a family of biodegradable biopolymers consisting of different hydroxyalkanoic acids that are synthesized and stored intracellularly by many microorganisms as carbon and energy reserves [3]. Owing to their biodegradability, biocompatibility, and sustainability, PHAs have been extensively investigated for industrial [4], biomedical [5,6], and environmental applications [7]. Recently, PHAs have gained interest in animal nutrition because their degradation products can serve as energy substrates for both intestinal microbiota and host tissues [8]. Unlike conventional OAs that are rapidly absorbed in the upper gastrointestinal tract, PHAs may provide a sustained release of biologically active metabolites throughout the digestive system, potentially enhancing their functional effects on gut health and nutrient utilization [9].

Among the various PHA polymers, poly(3-hydroxybutyrate) (PHB) has received the greatest attention. PHB can be depolymerized into water-soluble metabolites, including β-hydroxybutyrate, which may function as energy sources and microbial control agents within the gastrointestinal tract [10]. Such PHB can be produced via microbial fermentation under controlled conditions, offering a sustainable and economically viable feed ingredient [11]. Another PHA, poly(3-hydroxypropionate) (P3HP), also known as poly(3-hydroxypropionic acid), is biosynthesized by specific bacteria using 1,3-propanediol as a substrate [12]. Upon microbial or enzymatic degradation, P3HP releases 3-hydroxypropionic acid, a three-carbon organic acid that may participate in microbial fermentation processes and influence the gastrointestinal microbial ecosystem [12]. Dietary supplementation with PHB has been reported to enhance growth performance, stimulate innate immune responses, and improve digestive enzyme activities in several aquatic species [13,14]. However, Wang et al. [15] observed no significant effects on average daily gain, average daily feed intake, feed efficiency, or organ development in weaned piglets supplemented with 0.5% PHB. These inconsistent findings suggest that the physiological responses to polyhydroxyalkanoates (PHAs) may vary depending on the polymer type, animal species, and production stage.

Despite the potential biological relevance of P3HP, to date research has primarily focused on its microbial production [16], physicochemical properties [17], and industrial applications [18], whereas its use in monogastric nutrition remains largely unexplored. To the best of our knowledge, this is the first study to evaluate the potential of P3HP as a novel feed additive in pigs. Given the increasing interest in functional feed ingredients and the limited information available on the nutritional application of P3HP in livestock, this study aimed to investigate the effects of dietary P3HP supplementation on growth performance, nutrient digestibility, fecal nitrogen (N) retention and apparent N absorption, selected culturable fecal bacterial populations, and in vitro fecal fermentation in growing pigs. We hypothesized that dietary P3HP supplementation may exert dose-dependent effects on these parameters and provide new insights into its potential as a value-added bioproduct and novel functional feed additive for swine nutrition.

## 2. Materials and Methods

All experimental procedures involving animals were performed in accordance with the ethical guidelines established by the Institutional Animal Care and Use Committee (IACUC) of Dankook University, Republic of Korea, and were approved under protocol number DK-1-2610.

### 2.1. Characteristics of Poly(3-Hydroxypropionate)

P3HP (Product batch Number: 2603 30 HDPS) used in this study was synthesized and purified in the R&D laboratory of CJ CheilJedang (Suwon, Republic of Korea) using a proprietary production process. Purification of P3HP consists of multiple washes and solid–liquid separation and was confirmed using HPLC analysis. The polymer was characterized as a white solid [Method: GC-FID] containing 97.0–98.0% of P3HP with a moisture [Method: ASTM-E1868] content of 0.35% and a molecular weight [Method: GPC] (Mw, polystyrene-equivalent) of approximately 210 kDa. According to the analytical characterization, the polymer exhibited a melting temperature (Tm) of 80.43 °C, a crystallization temperature (Tc) of 37.17 °C, and a glass transition temperature (Tg) of −34.87 °C.

### 2.2. Experimental Facilities

The experiment was performed at the Dankook University Experimental Farm, Sejong, Republic of Korea. Animals were maintained in a double curtain-sided housing facility (10 × 18 ft) equipped with pit fans for minimum ventilation. The pigs were allocated to fully slated-floor pens (2.44 × 1.53 m) with a deep manure pit for waste collection. Each pen contained a nipple-type drinker and a single-space self-feeder, ensuring ad libitum access to drinking water and the experimental diets. Artificial lighting was provided for 12 h daily. During the first week, the room temperature was maintained at 30 °C and gradually reduced by 1 °C per week until stabilized at 24 °C. The facility was inspected three times a day (09:00, 13:00, 17:00) to monitor feed availability, water supply, and the health status of the animals.

### 2.3. Animals and Experimental Design

A 6-week growth trial was conducted using four groups of 25 pigs [100 head [Duroc × (Landrace × Yorkshire)] with initial BW 25.03 ± 1.70 kg]. The pens of pigs (2 barrows and 3 gilts/pen) were randomly allocated into four treatment groups with 5 replications/treatment. The dietary treatments were as follows: control (CON, basal diet—0%) and basal diet supplemented with 0.1, 0.2, and 0.3% of P3HP supplement. The basal diets (mash form) were formulated to meet or exceed the nutrient requirements of growing pigs as recommended by the NRC [19]. The ingredient composition and analyzed nutrient values of basal diet are shown in Table 1. The P3HP supplementation was top-dressed in basal diet and mixed using DK-801 feed mixture for uniform distribution.

### 2.4. Sampling and Laboratory Analysis

The BW of individual pigs was measured at the start of the trial and at the end of week 6. Average daily gain (ADG), average daily feed intake (ADFI), and feed conversion ratio (FCR) were determined on a pen basis over the entire experimental period. All pigs were weighed between 07:00 and 09:00 h before the morning feeding using an electronic scale with a precision of ±10 g. The ADG, ADFI, and FCR were calculated using the following equations.

$$ADG\left(g/day\right)=\frac{Final\;BW-Initial\;BW\left(g\right)}{Study\;period}$$

$$ADFI\left(g/day\right)=\frac{Total\;FI\left(g\right)}{Study\;period}$$

$$FCR=\frac{FI}{Weight\;Gain}$$

To determine the apparent total tract digestibility (ATTD) of dry matter (DM), nitrogen (N), and energy (E), chromium oxide (0.5%) was added to the basal diets as an indigestible marker at the beginning of week 5 and fed for 7 consecutive days. Representative feed samples were collected immediately after chromium oxide incorporation, sealed in zip-lock bags, and transported to the laboratory for subsequent analyses. At the end of week 6, fresh fecal samples were randomly collected from two pigs (one barrow and one gilt) per pen by gentle rectal stimulation. Fecal samples collected from the two pigs within each pen were pooled to obtain a single representative sample per pen. The pooled samples were placed in sterile, labeled plastic containers, transported to the laboratory, and stored at −15 °C until analysis. Prior to chemical analysis, fecal samples were dried in a forced-air oven at 80 °C for 72 h and ground to pass through a 1 mm sieve together with representative feed samples. Dry matter (DM) was determined according to AOAC Method 930.15, whereas nitrogen (N) was analyzed using AOAC Method 978.04. Chromium concentrations in feed and fecal samples were measured by UV absorption spectrophotometry (Shimadzu UV-1201, Kyoto, Japan). Gross energy (GE) was determined using a bomb calorimeter (Parr 6100, Parr Instrument Co., Moline, IL, USA). The apparent total tract digestibility (ATTD) was calculated using the following equation:

ATTD = {1 − [(Nf × Cd)/(Nd × Cf)] × 100}

Nf is the nutrient concentration in feces (% DM), Nd is the nutrient concentration in the diet (% DM), Cf is the chromium concentration in feces (% DM), and Cd is the chromium concentration in the diet (% DM). The apparent N absorption and fecal N excretion were analyzed using the following formula.

Apparent N absorption (g/d) = N intake × (Apparent N digestibility/100)

Fecal N excretion (g/d) = N intake − Apparent N absorption

At the end of week 6, fresh fecal samples were aseptically collected directly from the rectum of two pigs (one barrow and one gilt) randomly selected from each pen using sterile gloves. Fecal samples collected from the two pigs within each pen were pooled to obtain a single representative sample per pen. The pooled samples were transferred into sterile, labeled collection tubes, immediately placed in an ice box, and transported to the laboratory for the enumeration of selected culturable fecal bacteria. Briefly, 1 g of each fecal sample was homogenized in 9 mL of sterile 1% peptone broth (Becton, Dickinson, Sparks, MD, USA). Serial 10-fold dilutions were prepared using sterile 1% peptone solution, and appropriate dilutions were plated onto MacConkey agar (Difco Lab, Detroit, MI, USA) for the enumeration of *Escherichia coli* and Lactobacilli Medium III agar (DSMZ, Braunschweig, Germany) for the enumeration of *Lactobacillus*, respectively. MacConkey agar plates were incubated aerobically at 37 °C for 24 h, and *E. coli* colonies were identified based on their characteristic bright pink to red coloration and round colony morphology. Lactobacilli Medium III agar plates were incubated anaerobically at 39 °C for 48 h in an atmosphere containing 85% N_2_, 10% CO_2_, and 5% H_2_. Following incubation, bacterial colonies were enumerated, and the results were expressed as log_10_ colony-forming units (CFU)/g before statistical analysis.

At the end of week 6, fresh fecal samples were collected from two randomly selected pigs (one barrow and one gilt) in each pen by gentle rectal massage. Approximately 300 g of feces from the two pigs within each pen was thoroughly mixed to obtain one representative fecal sample per pen. Concurrently, urine samples were also collected using a plastic bucket. Later, the fecal and urine samples were mixed, poured into 2600 mL airtight plastic containers sealed with rubber stoppers and aluminum caps, and incubated at room temperature (25 °C) for 7 days to allow in vitro fecal fermentation. After the fermentation period, approximately 100 mL of headspace sample was withdrawn to facilitate air circulation. The container was then re-sealed and gently shaken for approximately 30 s to disrupt the crust formed on the surface. Finally, the concentrations of ammonia (NH_3_), hydrogen sulfide (H_2_S), carbon dioxide (CO_2_), methyl mercaptan, and acetic acid produced during in vitro fecal fermentation were determined using a multifunctional MultiRAE Lite gas detector (Model PGM-6208, RAE Systems, San Jose, CA, USA).

### 2.5. Statistical Analysis

All statistical analyses were performed using SAS software (version 9.4; SAS Institute Inc., Cary, NC, USA). Data were analyzed as a completely randomized design using analysis of variance (ANOVA), with pen considered the experimental unit. The statistical model included dietary treatment as the fixed effect. Orthogonal polynomial contrasts were used to evaluate the linear, quadratic, and cubic responses to increasing dietary P3HP supplementation. Prior to analysis, data were assessed for normality using the Shapiro–Wilk test and for homogeneity of variance using Levene’s test. When a significant treatment effect was detected, mean comparisons were performed using Tukey’s honestly significant difference (HSD) test. Statistical significance was declared at *p* < 0.05, whereas 0.05 ≤ *p* < 0.10 was considered to indicate a statistical trend.

## 3. Results and Discussion

The present study demonstrated that dietary supplementation with 0.2% P3HP showed a tendency (*p* < 0.10) to improve growth performance and nutrient utilization in growing pigs, as indicated by the linear and quadratic responses observed for final body weight, ADG, DM digestibility, N digestibility, and apparent N absorption (Table 2 and Table 3). The numerical responses were mainly observed at the 0.2% inclusion level, suggesting that moderate supplementation may be sufficient to elicit potential beneficial effects. However, as this is the first study investigating P3HP as a dietary feed additive in pigs, further studies are required to confirm the consistency and biological significance of these responses. P3HP belongs to the polyhydroxyalkanoate (PHA) family [12], which consists of biodegradable biopolymers with diverse physicochemical properties. Previous studies have suggested that PHA-related compounds may undergo microbial or enzymatic degradation under certain biological conditions, potentially generating smaller metabolites [17]. Although 3-HP is structurally related to propionic acid, an organic acid commonly used in animal nutrition for its preservative and antimicrobial properties [20,21], structural similarity alone does not confirm identical biological activities. Therefore, the potential contribution of P3HP-derived metabolites to the observed improvements in nutrient utilization should be considered a possible mechanism that requires further experimental validation.

The tendency for increased DM and N digestibility suggests that P3HP supplementation may influence digestive efficiency and nutrient availability. OA and short-chain fatty acids have been reported to improve nutrient utilization through multiple mechanisms, including modulation of intestinal pH, enhancement of epithelial barrier function, stimulation of digestive enzyme activity, and improvement of nutrient absorption conditions [22,23,24]. Unlike conventional OAs, which are rapidly absorbed in the upper gastrointestinal tract, P3HP may potentially provide a different pattern of metabolite availability due to its polymeric structure and degradation characteristics. Although this proposed mechanism remains speculative, a gradual release of degradation products may represent one possible explanation for the observed improvements in nutrient digestibility. Future studies directly evaluating P3HP degradation kinetics and metabolite production are necessary to clarify this hypothesis. Despite the observed improvements in growth performance and nutrient utilization, dietary P3HP supplementation did not significantly affect fecal *Lactobacillus* or *Escherichia coli* counts (Table 4). This result indicates that the supplementation levels used in the present study were insufficient to induce detectable changes in these culturable fecal bacterial populations. Since fecal bacterial enumeration represents only a limited assessment of the gastrointestinal microbial ecosystem, future studies using comprehensive microbiome and metabolomic approaches are needed to determine whether P3HP influences broader microbial community structure or function. Similarly, no significant differences were observed in gas production during in vitro fecal fermentation (Table 5). We speculate that the numerical improvements in N digestibility and apparent N absorption may have reduced the amount of undigested nitrogenous substrates available for microbial fermentation after excretion [25]. However, these improvements were likely insufficient to produce measurable changes in fecal gas production. The underlying reason for the lack of response remains unclear, and further studies are warranted to elucidate the mechanisms by which P3HP supplementation influences fecal fermentation and gas production. Overall, dietary supplementation with P3HP showed linear or quadratic trends for several variables while most of the principal outcomes did not reach statistical significance. Furthermore, the relatively limited number of independent pen replicates per treatment and the absence of urinary nitrogen measurements limited the comprehensive evaluation of nitrogen utilization. Only two groups of culturable fecal bacteria were examined, and P3HP degradation and the in vivo release of 3-hydroxypropionic acid were not directly measured. Furthermore, intestinal morphology, barrier function, digestive enzyme activity, microbial metabolism, and the safety and tolerance of P3HP supplementation were not systematically evaluated. Therefore, further studies with larger sample sizes and more comprehensive physiological and mechanistic assessments are warranted to validate the biological effects and clarify the mode of action of P3HP in growing pigs.

## 4. Conclusions

Dietary supplementation with 0.1–0.3% P3HP did not produce significant effects on growth performance, nutrient digestibility, selected culturable fecal bacterial counts, or in vitro fecal fermentation. Nevertheless, several performance- and nutrient utilization-related variables showed favorable numerical trends, with the greatest responses observed at the 0.2% supplementation level. As the first investigation of P3HP as a dietary feed additive in pigs, we believe that this study would provide a foundation for future research aimed at elucidating its mechanisms of action and identifying the optimal supplementation strategy for maximum benefits.

## Figures and Tables

**Table 1:** Composition of growing pig diets (as-fed basis).

Ingredients (%)	
Corn	75.56
Soybean meal	18.22
Tallow	0.65
Molasses	2.00
Mono-Dicalcium Phosphate	1.10
Limestone	0.84
Lysine (78%)	0.50
Methionine (98%)	0.06
Threonine (98%)	0.17
Tryptophan (98%)	0.02
Arginine (98.5%)	0.18
Salt	0.20
Mineral mix ^1^	0.20
Vitamin mix ^2^	0.20
Choline (25%)	0.10
Total	100.00
Calculated value	
Dry matter, %	88.82
Crude protein, %	16.00
Metabolizable Energy, kcal/kg	3300
Calcium, %	0.66
Phosphorus, %	0.56
Lysine, %	1.12
Methionine, %	0.32
Threonine, %	0.72
Tryptophan, %	0.19
Crude Fat, %	3.55
Crude Fiber, %	2.20
Crude Ash, %	4.11

^1^ Supplied per kilogram of diet: 100 mg Fe (from ferrous sulfate), 17 mg Cu (from copper sulfate), 17 mg Mn (from manganese oxide), 40 mg Zn (from zinc oxide), 0.5 mg I (from potassium iodide), and 0.3 mg Se (from sodium selenite). ^2^ Supplied per kilogram of diet: vitamin A, 10,800 IU; vitamin D_3_, 4000 IU; vitamin E, 40 IU; vitamin K_3_, 4 mg; vitamin B_1_, 6 mg; vitamin B_2_, 12 mg; vitamin B_6_, 6 mg; vitamin B_12_, 0.05 mg; biotin, 0.2 mg; folic acid, 2 mg; niacin, 50 mg; and D-calcium pantothenate, 25 mg.

**Table 2:** The effects of dietary P3HP supplementation on growth performance and nutrient digestibility in growing pigs ^1^.

Items	0%	0.1%	0.2%	0.3%	SEM ^2^	*p*-Value ^3^		
						Linear	Quadratic	Cubic
Growth Performance								
Body weight, kg								
Initial	25.03	25.04	25.04	25.03	0.02	0.866	0.615	0.821
Week 6	52.25	53.95	54.09	53.72	0.53	0.081	0.075	0.657
Overall								
ADG, g	649	688	692	682	13	0.092	0.074	0.706
ADFI, g	1413	1439	1443	1435	24	0.536	0.475	0.934
FCR	2.177	2.091	2.087	2.105	0.033	0.159	0.136	0.693
Nutrient digestibility								
Week 6								
Dry matter, %	72.82	74.25	74.47	74.04	0.52	0.111	0.087	0.812
Nitrogen, %	69.13	70.87	71.11	70.61	0.58	0.082	0.064	0.772
Energy, %	71.72	73.23	73.46	72.91	0.64	0.196	0.120	0.862

^1^ Abbreviation: Control (CON, basal diet—0%) and basal diet supplemented with 0.1, 0.2, and 0.3% of P3HP supplement. ^2^ Standard error of means. ^3^ *p* < 0.05 was set as statistically significant and 0.05 < *p* ≤ 0.10 was set as trends.

**Table 3:** The effects of dietary P3HP supplementation on apparent N absorption and fecal N excretion in growing pigs at week 6.

Items	0%	0.1%	0.2%	0.3%	SEM ^2^	*p*-Value ^3^		
						Linear	Quadratic	Cubic
DM intake, g/d	1264.67	1289.26	1294.93	1287.59	25.88	0.457	0.476	0.953
Nitrogen intake, g/d	32.49	33.18	33.22	32.89	0.69	0.623	0.370	0.913
Apparent N absorption, g/d	22.46	23.51	23.62	23.22	0.51	0.046	0.481	0.889
Fecal N excretion, g/d	10.03	9.67	9.60	9.67	0.42	0.281	0.418	0.971

^2^ Standard error of means. ^3^ *p* < 0.05 was set as statistically significant and 0.05 < *p* ≤ 0.10 was set as trends.

**Table 4:** The effects of dietary P3HP supplementation on selected culturable fecal bacterial counts in growing pigs at week 6.

Items, log_10_ CFU/g	0%	0.1%	0.2%	0.3%	SEM ^2^	*p*-Value ^3^		
						Linear	Quadratic	Cubic
*Lactobacillus*	6.53	6.56	6.57	6.54	0.03	0.564	0.230	0.739
*E. coli*	5.14	5.10	5.12	5.13	0.05	0.903	0.650	0.825

^2^ Standard error of means. ^3^ *p* < 0.05 was set as statistically significant and 0.05 < *p* ≤ 0.10 was set as trends.

**Table 5:** The effects of dietary P3HP supplementation on gas concentrations after in vitro fecal fermentation in growing pigs at week 6.

Items, ppm	0%	0.1%	0.2%	0.3%	SEM ^2^	*p*-Value ^3^		
						Linear	Quadratic	Cubic
NH_3_	5.40	5.15	5.00	5.25	0.54	0.805	0.645	0.902
H_2_S	4.45	4.53	5.38	4.43	0.43	0.687	0.246	0.197
Methyl mercaptans	8.25	8.50	8.40	8.15	0.67	0.895	0.713	0.947
Acetic acid	8.90	8.75	8.50	8.65	0.65	0.733	0.819	0.864
CO_2_	13,620	13,700	13,670	13,580	428	0.938	0.844	0.979

^2^ Standard error of means. ^3^ *p* < 0.05 was set as statistically significant and 0.05 < *p* ≤ 0.10 was set as trends.

## Data Availability

The datasets generated and/or analyzed during the current study are available within the article. Additional information is available from the corresponding author upon reasonable request.
